# Congenital Anomalies of the Nose: From Embryologic to Surgical Treatment

**DOI:** 10.7759/cureus.89397

**Published:** 2025-08-05

**Authors:** Theodora-Eleftheria Deftereou, Vaia Karapepera, Stergios Lialiaris, George Fotiadis, Konstantinos Chaidas, Michail Katotomichelakis

**Affiliations:** 1 Laboratory of Histology-Embryology, Democritus University of Thrace, Alexandroupolis, GRC; 2 Ear, Nose, and Throat (ENT) Clinic, General Hospital of Ioannina “G. Chatzikosta”, Ioannnina, GRC; 3 Ear, Nose, and Throat (ENT) and Head and Neck Surgery, University General Hospital of Alexandroupolis, Democritus University of Thrace, Alexandroupolis, GRC

**Keywords:** birth defect, embryology of nose, facial development, nasal congenital disorders, organogenesis

## Abstract

The nose, a facial organ, not only plays a crucial role in olfaction and respiration but also has a major impact on the overall anatomy of the face. However, congenital anomalies affecting the nose can be challenging as they require both functional impairments and cosmetic concerns. Facial maldevelopments during the early embryonic period induce a wide-ranging nasal deformity. Understanding the etiology of these anomalies is essential for healthcare professionals involved in diagnosis, treatment, and management. Based on current literature, this review aims to present the spectrum of congenital disorders of the nose and a summary of their embryologic etiology, clinical presentations, and therapeutic interventions.

## Introduction and background

Congenital nasal anomalies are extremely rare, impacting one in 20.000-40.000 live births, and encompass a wide array of conditions, ranging from mild to severe presentations [1]. These anomalies that have embryologic origin may involve nasal structure, shape, size, and function. Common anomalies include cleft lip and palate, nasal septal defects, nasal pyriform aperture stenosis, choanal atresia, nasal dermoid cysts, and nasal hemangiomas, among others. Each anomaly presents unique challenges and requires tailored management strategies [2].

Nasal embryologic development is a complex and tightly regulated process that begins early in gestation and continues throughout fetal development. Understanding the intricate steps involved in the formation of the nasal structures is crucial for comprehending the etiology of congenital nasal anomalies and guiding clinical management. Nasal embryonic development can be broadly divided into several stages.

Formation of nasal placodes

During the fourth week of gestation, two bilateral ectodermal thickenings, nasal placodes, form on the frontonasal process, one of the five facial primordia, which is derived from the ectoderm. These placodes undergo invagination to form the nasal pits on each side, marking the initial stage of the nasal cavities [3].

Proliferation and fusion

As development progresses, the nasal pits deepen and undergo medial and lateral growth. Concurrently, the medial and lateral nasal prominences, derived from the frontonasal process, develop on either side of the nasal pits. These prominences contribute to the formation of the nasal dorsum and lateral nasal walls [3].

Fusion of facial processes

Around the seventh week of gestation, the lateral nasal prominences fuse with the maxillary processes, giving rise to the ala nasi and the lateral border of the nostril [3]. Failure of fusion can result in midline defects such as a cleft lip and palate. In the eighth gestational week, the medial nasal prominences migrate towards the midline and eventually fuse, forming the intermaxillary segment, the precursor of the nasal septum. Nasomedial processes also fuse with maxillary prominences to form the philtrum [4]. The glabella, the area between the eyebrows and above the nasal root, develops directly from the frontonasal process [2].

Differentiation and maturation

During the later stages of embryonic development, the nasal structures undergo further differentiation. By the end of the 14th week, facial formation is complete [2].

## Review

Nasal maldevelopments overview

Over the years, various classification systems have been proposed to categorize these anomalies based on their anatomical characteristics, etiology, and associated cranio-facial deformities [5,6]. In 2004, Losee et al. [7] developed a classification system specifically for congenital nasal anomalies, categorizing them into four main groups: Type I: hypoplasia and atrophy; Type II: hyperplasia and duplications; Type III: clefts; and Type IV: neoplasms and vascular anomalies. More recently, Fijalkowska and Antoszewski suggested that vascular anomalies should not be included in congenital nasal deformities [1]. Based on the Losee classification scheme, this review aims to explore various congenital nasal anomalies, their diagnosis, and the surgical treatment modalities available for their management.

Type I: Hypoplasia and atrophy

*Total arrhinia* is defined by Rosen in 1963 as an exceedingly rare congenital anomaly characterized anatomically by the lack of the entire nose, the nasal cavities, and the olfactory apparatus [8,9]. To date, fewer than 50 cases of total arrhinia have been reported in the literature [10]. Defects in the development of both the nasal placodes and, consequently, the medial and lateral nasal processes, as well as the early fusion of nasomedial prominences, have been suggested as causative factors for total arrhinia [10-12]. Arrhinia has been associated with other facial dysmorphias such as hypertelorism, microphthalmia, colobomas, and nasolacrimal duct abnormalities. In addition, specific de novo mutations have recently been proposed as contributing factors [13]. Ultrasound diagnosis can be made prenatally, although more reported cases have been diagnosed after birth [12].

Arrhinia can be classified into three groups of malformations: total arrhinia, hemi-arrhinia (often referred to as hemi-nasal agenesis), and proboscis lateralis (PL). Mazzola et al. also described two additional subtypes of heminasal aplasia: the half nose and the half nose with PL [14]. Half-nose, also known as partial arrhinia, is a rare congenital defect that affects unilaterally the external nose and the internal nasal cavity [15], forming one nostril and one olfactory tract [16]. Heminasal aplasia is believed to result from the absence of one of the nasal placodes in early embryonic development [7,17]. Clinically, heminasal aplasia is rarely observed as an isolated anomaly; in most reported cases, it is accompanied by other facial abnormalities [18]. Among them, PL, discussed below, is the most common [16].

*Partial nasal hypoplasia* is typically presented with a visibly smaller or misshapen nose. The degree of hypoplasia can vary widely. In some cases, it may only involve the external nose, resulting in a cosmetic issue, while in others, it can affect the internal structures, leading to functional impairments such as breathing difficulties. Isolated absence of the dorsum, non-appearance of the columella, absence of the septal cartilage, and absence of the tip of the nose are some of the most frequent abnormalities that, as entities, have a distinct origin on a defective medial nasal process [19]. Treatment of rhinophyma aims for surgical correction with the creation of a new nasal cavity and external nose, which is usually staged. The earliest stage for surgery is debated, although it is usually performed at the age of 5 years, followed by a final intervention near puberty. Most neonates adapt to oral breathing, but in severe cases, where adaptation is not prompt, tracheostomy is deemed appropriate until the staged reconstruction. In case of heminasal aplasia or partial nasal hypoplasia, rhinoplasty, often using an oblique forehead flap, is required [20].

*Nasal cavity atresia*,** **commonly referred to as choanal atresia (CA), is a congenital condition in which the posterior nasal airway (choana) is blocked by bony, membranous, or mixed membranous-bony tissue, preventing normal airflow between the nasal cavity and the nasopharynx. The estimated incidence is approximately one in 5,000 newborns [21]. It’s a well-known disorder first described by Roederer in 1755 [3]. Early in fetal development, the oronasal membrane is formed to separate the nasal cavity from the oral cavity. This hymen normally breaks down around the seventh week to establish an open communication between the two spaces. Failure of this membrane to fully dissolve, or abnormal development of surrounding bony structures, leads to CA [7,19]. However, previous theories suggested that CA can be caused by persistence of the buccopharyngeal membrane, by abnormal location of mesoderm-forming adhesions in the nasochoanal area, or by false migration of neural crest cells [3]. The majority of cases are unilateral, the right side is commonly affected, and the condition displays a female predominance (female-to-male ratio, 2:1) [3,4]. Signs and symptoms of nasal obstruction in newborns are the clinical manifestations of CA, including noisy breathing, feeding difficulties, and persistent mucoid rhinorrhea. In cases of bilateral CA, acute respiratory distress may be present [2]. CA is frequently associated with isolated malformations such as coloboma, heart anomalies, and ear deformities, or it may occur as part of a syndrome, most commonly CHARGE syndrome [21]. Unilateral CA is not associated with acute respiratory distress; thus, its diagnosis is usually established later. In contrast, bilateral CA in a newborn requires prompt diagnosis and management with airway stabilization by inserting an oral airway to break the seal formed between the tongue and the palate, which can be well tolerated for several weeks. Several techniques to repair CA have been reported, including transnasal, transeptal, transpalatal, endoscopic nasal or retropalatal, and combined transoral-transnasal approach, but none of them has gained universal acceptance [22].

Congenital nasal pyriform aperture stenosis (CNPAS) is a rare developmental deformity that affects nasal obstruction in neonates due to abnormal narrowing of the nasal pyriform aperture, the bony opening in the skull through which the nasal cavity communicates with the external environment [2,23]. CNPAS was first described by the radiologist Belden in 1989 [24]. It has been suggested that during the fourth to eighth week of gestation, a hypertrophy of maxillary ossification at the nasal process of the maxilla results in CNPAS [3,25]. The clinical presentation seems to be common with CA [21]. CNPAS can be sporadic or occur in association with other craniofacial abnormalities. For instance, it is often found in conjunction with holoprosencephaly, a severe congenital malformation of the brain, which also involves defects in the Sonic Hedgehog (SHH) signaling pathway [2]. Mild CNPAS can be managed with insertion of silastic stents within the nasal cavity in combination with the use of local decongestants. On the other hand, in cases of moderate or severe stenosis, surgical treatment is required involving pyriform aperture enlargement via endo-oral sublabial or transnasal approach [26].

**Figure 1:**
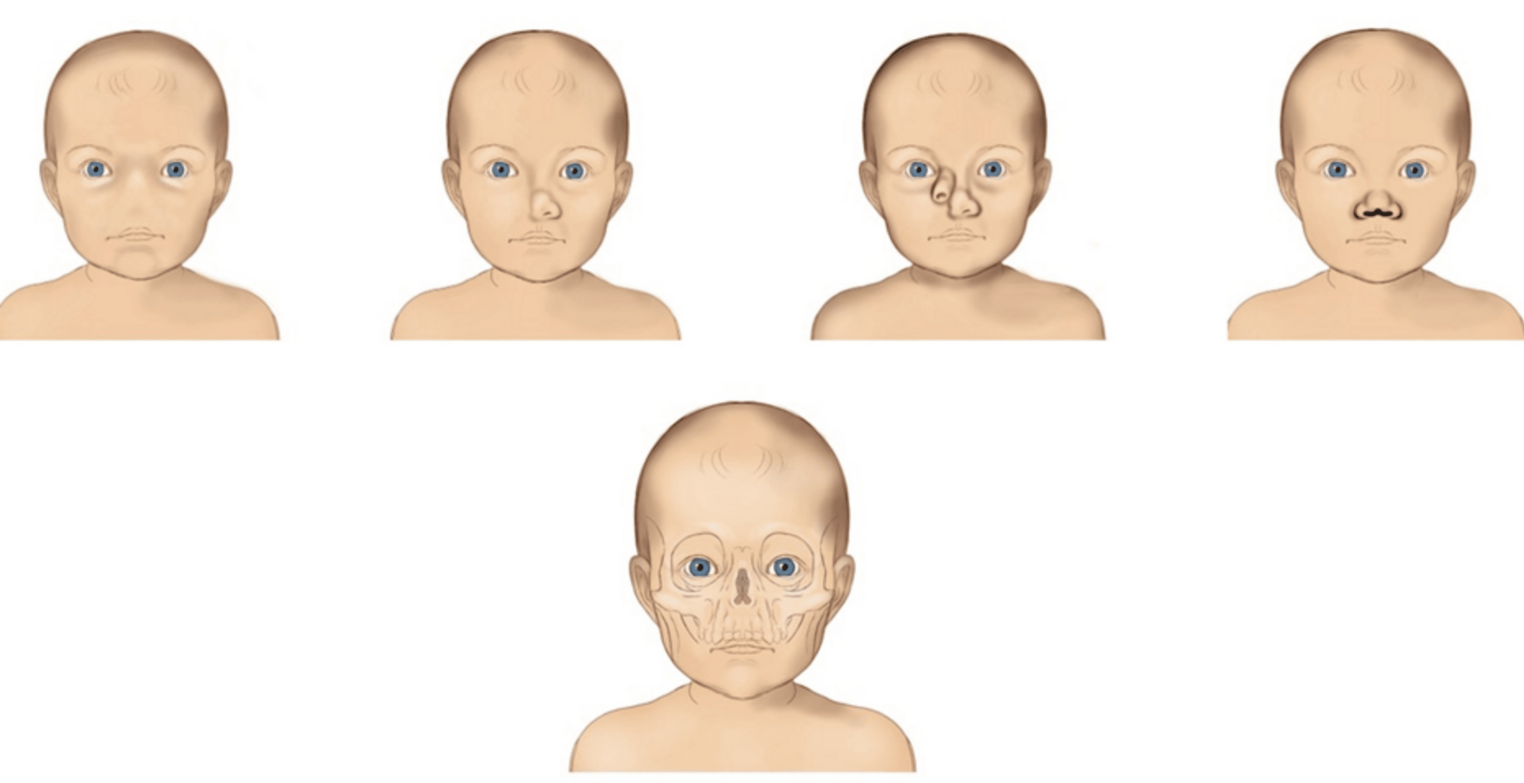
Schematic representation of various Type 1 nasal congenital disorders. Image credit: Vaia Karapepera.

Type I nasal congenital disorders are shown in Figure 1.

Type II: Hyperplasia and duplications

*Polyrrhinia*, an extremely unusual nasal abnormality, has been at first described by Erich in 1961 as the presence of two noses, each one having two nostrils and two nasal cavities [27]. A double nose is suggested to result when each nasal placode gives rise to two nasal pits, forming four nasal sacs presented horizontally. The two medial sacs were between the nasal laminae, preventing the laminae from being fused, forming one nasal septum. As a result, two septa, four nostrils, and four nasal cavities were formed [7,28]. To date, only a few cases of polyrrhinia have been reported in the literature. Surgical management consists of excision of the medial halves of each nose.

*Supernumerary nostril*, also known as an accessory nostril, is a congenital condition in which one or more extra nostrils are formed in addition to the normal pair. As polyrrhinia, supernumerary nostril is included in duplication nasal deformities [29], but differs from this defect [30]. Accessory nostril is typically located close to the normal nostril on the same side, having its nasal passage, though it might be rudimentary or non-functional. Supernumerary nostril seems to be found more often on the left side [29]. It has been proposed that during early fetal development, an accessory nasal pit forms lateral to the nasal lamina, resulting in the development of an additional nostril [31,32]. Management of supernumerary or accessory nostrils consists of early surgical excision of the fistulous or blind tract. Fistulo-rhinostomy may be required when the defect cannot be covered by a local skin flap.

PL is an extremely rare deformity, defined as an epithelialized, tubular, or trunk-like appendage resembling an elongated nose, with a birth prevalence estimated between 1 in 100,000 and 1 in 1,000,000 live births [33]. It typically occurs unilaterally, near the medial canthus [34]. It is often not connected to the nasal cavity, and the normal nostril or nasal structures on that side may be underdeveloped or absent. Thus, PL is commonly associated with heminasal aplasia, CA, and paranasal sinus hypoplasia as well as with other craniofacial anomalies, such as abnormalities in the orbit [2,33]. According to Martin et al., heminasal aplasia with PL is caused when a segregation of the medial nasal prominence occurs, forming two sections. At the same time, a defective fusion of maxillary and lateral nasal processes can be manifested as PL [19]. Surgical treatment involves complete excision at the base of the proboscis, followed by staged reconstruction beginning in adolescence [35]. The main differences between PL and supernumerary nostrils are summarized in Table 1.

**Table 1:** Differences between proboscis lateralis and supernumerary nostril.

Feature	Proboscis lateralis	Supernumerary nostril
Incidence [29,30,32,33]	Extremely rare	Extremely rare, even less common than proboscis lateralis
Appearance [30,33]	Trunk-like, tubular appendage	An extra nostril close to the normal one, usually open
Location [29,30,33]	Lateral side of the face	Adjacent to one of the primary nostrils
Functionality [29,30]	Not functional for breathing	May or may not be functional
Connection to the nasal cavity [29,30]	Often no connection	May have a rudimentary connection to the nasal cavity
Associated anomalies [29,33]	Often associated with craniofacial defects	Can be isolated or with minor facial deformities
Embryologic cause [31,32,34]	Abnormal development of nasomedial prominence	Accessory nasal pit

**Figure 2:**
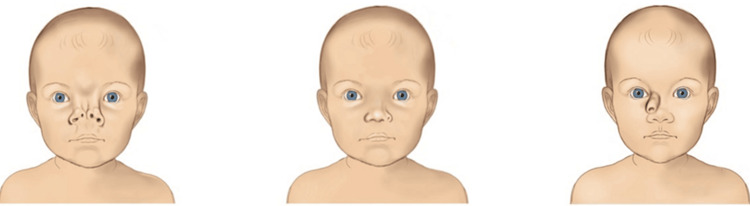
Schematic representation of various Type II nasal congenital disorders. Image Credit: Vaia Karapepera.

Figure 2 presents the Type II malformations.

Type III: Nasal clefts

Nasal clefts are rare congenital malformations often presenting as part of a broader spectrum of craniofacial anomalies, significantly impacting aesthetics and function. They range from isolated midline clefts to more complex presentations, and their incidence rates are significantly lower than other types of craniofacial clefts. Birth prevalence is estimated to be around 1.43 to 4.85 per 100,000 births, accounting for almost 2% of all craniofacial clefts [36]. Tessier’s classification system is widely used, describing the clefts according to their anatomical location on a numeric scale from 0 to 14. Facial clefts involving the nose have been defined as Tessier nos. 0, 1, 2, and 3, and their cranial extensions have been described as Tessier nos. 14, 13, 12, and 11, respectively [20].

*Tessier no. 0 *is a rare midline deformity restricted to the nose and clinically manifested by the nasal division into two equal parts below the orbital level. It is more often involving the forehead extending superiorly toward the nasofrontal junction, as well as affecting the upper lip, presented as a bifid philtrum. This deformity is widely known as Tessier no. 0/14 cleft and is typically caused by a failure in the fusion of the medial nasal prominences during early embryonic development [2,37]. The severity of this malformation can vary widely, ranging from subtle nasal asymmetry to extensive facial disruptions that implicate the nasal cartilage, bone, and soft tissue. Common features may include the absence of the columella or nasal septum, and a bifid nasal tip [37].

*Tessier no. 1*typically manifests as a paramedian cleft affecting the dome of the nose and the nasal ala, resulting in the separation of the alar lobule from the nasal tip. This deformity is also known as nasoschisis. Nasal ala could be found flattened, and the alar base widened [7]. The cleft can be extended below involving the cupid’s bow as well as toward the medial brow or even to the medial canthus, demonstrating as Tessier 1/13 cleft. Bony involvement may be present, affecting the central or lateral incisors, the nasal bones, the pyriform aperture, or even the frontal process of the maxilla. Defective nasal processes fusion or disrupted neural crest cells migration in early nasal embryogenesis have been proposed as possible causative factors [2,37,38]. 

*Tessier no. 2* is another rare craniofacial anomaly affecting the nasomaxillary area. This disorder is a notch located in the middle third of the nasal rim, lateral to Tessier no. 1 [39]. Typically, the cleft is located between the tail of the alar cartilage and the alar base. Tessier No. 2/12 extends from the alar rim to the upper lip, lateral to the philtral column, and cranially through the frontal process of the maxilla [2]. Bony involvement may be present at the lateral incisor and could extend through the pyriform aperture, affecting even the ethmoid sinus, which may be enlarged, and reaching the medial orbital rim [7,39]. Hypertelorism is a frequently associated malformation caused by ethmoid sinus enlargement. A defective fusion between the nasal processes and the maxillary process has been considered the cause of this disorder.

*Tessier no. 3 *is the most often deformity among all nasal clefts, primarily located along the naso-orbital line. In this disorder, the third part of the nasal ala is mainly affected. The cleft can extend from the base of the nasal ala toward the medial canthus and downward to the Cupid’s bow, presented as Tessier no. 3/11. Common features include notching of the nostril, displacement of the medial canthus, and, occasionally, associated orbital deformities such as microphthalmia and anophthalmia. On the upper lip, the cleft disrupts the philtral ridge and Cupid’s bow, resulting in asymmetry or complete distortion. The nasolacrimal system may be involved in cases where bony implication is presented [2,37]. Osseous involvement may affect the area between the lateral incisor and canine, the palate, the pyriform aperture, and even the medial wall of the maxillary sinus [39]. The oro-naso-ocular cleft is considered to result from defective fusion among the lateral nasal, medial nasal, and maxillary prominences. Another theory suggests that the deformity may be caused by insufficient invagination of the nasolacrimal groove [37].

**Figure 3:**
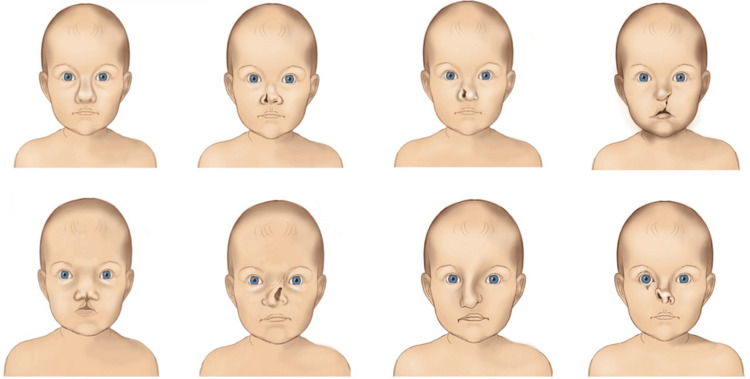
Schematic representation of various Type III nasal congenital disorders. Image Credit: Vaia Karapepera.

Considering the underlying complexity of nasal clefts, a *one-size-fits-all* therapeutic approach is not indicated. Every cleft should be carefully evaluated to determine the degree of pathology before treatment planning. Nasal clefts require surgical reconstruction, with their extent depending on the severity. Surgery consists of open, semi-open, or closed septorhinoplasty and can be classified into primary, intermediate, and definitive, based on the timing of intervention. The optimal time for definitive repair varies depending on the severity of underlying pathology and associated craniofacial abnormalities [40,41]. All the aforementioned disorders are shown in Figure 3.

Type IV: Neoplasms and vascular anomalies

Congenital masses of the nose are a heterogeneous group of rare developmental anomalies that appear as abnormal tissue growths in or around the nose [42]. These masses originate during embryogenesis and often represent remnants of tissue that failed to involute or fuse properly. They can vary in presentation, size, location, and complexity and are broadly categorized into nasal dermoids, gliomas, and encephaloceles, among others. Early diagnosis and appropriate treatment are crucial due to the potential for airway obstruction, recurrent infections, or intracranial communication.

*Nasal dermoids* were first described by Cruvelier in 1817 [43]. They are the most often congenital deformities of the midline nasal structures, representing about 1%-3% of all dermoid cysts and 4%-12% of all congenital nasal masses [44], with a clear male predilection [45]. These anomalies originate due to incomplete regression of dural diverticulum - a transient outpouching of dura mater that reaches out through the foramen cecum to the prenasal space, approaches the tip of the nose, and throws back into the cranium [21]. A faulty regression of dural diverticulum may pull ectodermal elements into the prenasal space, resulting in nasal dermoids [46]. Nasal dermoids may present clinically as a cyst, if ectodermal elements are trapped beneath the skin; as a sinus, if the ectoderm withdraws along the dura while retaining communication with the skin; or as a fistula. They may involve only superficial tissues or extend deeper into the nasal bones, cartilages, and, in some cases, the anterior cranial fossa [45]. Nasal dermoids may be found anywhere along the course from the anterior cranial fossa to the nasal columella, with the most frequent site by far being the nasal dorsum, while approximately one in three cases appears to be intranasal [4,21]. Histologically, nasal dermoids contain skin tissues or dermal appendages such as hair follicles and sebaceous or sweat glands, and are covered by keratinized stratified squamous epithelium [21]. Biopsy of nasal dermoids is contraindicated, and the diagnosis depends on clinical and radiological findings (CT or MRI). Treatment involves complete cyst and sinus tract excision to avoid recurrence. In case of intracranial extension, dermoids should be differentiated from encephaloceles, and a combined craniofacial approach is required [47].

*Nasal gliomas*, also known as nasal glial heterotopia, were first described by Reid in 1852. They are benign and extremely rare congenital deformities that represent about 5% of all neuroglial heterotopias [48]. Despite their name, they are not true gliomas as they are non-proliferative and lack invasive growth typical of malignant gliomas. They result from developmental anomalies during embryogenesis. These lesions are thought to arise due to the abnormal closure of the anterior neuropore; as a result, a herniation or separation of tissue through the fonticulus frontalis or foramen cecum occurs. They consist of mature glial tissue, mainly astrocytes. Surrounding connective tissue and occasional blood vessels may also be present. They are mostly located outside the cranial cavity, with the nasal dorsum being the most frequent site [49], whereas intranasal gliomas are usually found on the lateral wall of the middle turbinate [50]. Nasal gliomas typically present as firm, non-compressible masses in or near the nose, sometimes at birth or during early infancy [2,51]. Differential diagnosis should include nasal dermoids, nasal encephaloceles, and nasal polyposis [2,50]. Biopsy is contraindicated. MRI scan provides optimal imaging of the soft tissue components, whereas a CT scan can identify potential skull base defects. Management consists of complete surgical excision to avoid recurrence. Extranasal gliomas can be managed through standard surgical excisions, while intranasal masses can be approached through lateral rhinotomy. In case of gliomas with intracranial extension, neurosurgical intervention is required [52].

*Nasal encephaloceles* are congenital malformations where intracranial contents (e.g., meninges, cerebrospinal fluid, and brain tissue) herniate through a defect in the skull base into the nasal region [2]. As nasal dermoids, encephaloceles result from the extension of a dural diverticulum through the fonticulus frontalis or foramen cecum [21]. The estimated incidence is approximately one of every 4,000 live births, and they show no clear sex predisposition [53]. Nasal encephaloceles are classified into two types based on their anatomical location relative to the nasal cavity. The first type, accounting for 15%, is located anteriorly and is referred to as sincipital encephaloceles. In this case, encephaloceles, which are protruding through the fonticulus frontalis, are distinguished as nasofrontal, and they are the commonest. Nasoethmoidal encephaloceles are defined by herniation through the foramen cecum, while nasoorbital encephaloceles occur through the medial canthus [54]. Sincipital encephaloceles clinically present as soft, pulsatile, bluish masses located at the glabella or on the nose [7,54]. On the other hand, nearly 75% of encephaloceles are occipital [53]. These are also known as basal encephaloceles, herniating through the cribriform plate into the nasal cavity and typically presenting as intranasal masses, which are often externally invisible. Basal encephaloceles are intranasal, smooth, compressible, pink, polypoid-appearing lesions [2,54]. They have been associated with complications like nasal airway obstruction and meningitis, as they are not externally apparent [55]. Histologically, nasal encephaloceles demonstrate similarities to gliomas, consisting of neural and fibrous tissue, whereas glial cells, cerebral tissue, and ependymal cells may also be present. Skin or nasal mucosa may cover the mass, depending on the site. The identification of leptomeninges is pathognomonic for encephaloceles [46]. Microcephaly, microopthalmia, hydrocephalus, corpus callosum dysgenesis, and interhemispheric lipoma may be manifested as associated congenital lesions [53]. Biopsy is strongly contraindicated due to the risk of infection and meningitis. Both MRI and CT scans are valuable to assess soft tissue herniation and bony defects, respectively. Treatment involves surgical excision with repair of the bony defect. Endoscopic transnasal ± transoral approach can be adequate, but craniotomy is often necessary to gain better access [56].

Taking into consideration the similarities that nasal dermoids, gliomas, and encephaloceles display, as well as the fact that they share a common embryologic pathogenesis, the differential diagnosis should be based on clinical and histological examination.

*Nasal teratomas *are exceedingly rare congenital lesions that belong to the family of germ cell tumors. They are generally benign and non-invasive, exhibiting slow growth. Teratomas of the head and neck represent about 5% of total teratomas, and the nasopharynx is by far the most frequent location, whereas the nasal cavity is remarkably unusual [57]. They also lack gender predominance, in contrast to teratomas in other sites [58]. These deformities are characterized by the presence of tissues foreign to the site of origin, coming from all three germ layers: ectoderm, mesoderm, and endoderm [59]. These tumors arise due to aberrations in embryologic development, specifically involving the improper migration or differentiation of pluripotent cells [60]. However, another theory supports that teratomas come from remnants of the primitive node, explaining the high frequency of midline structures where teratomas occur [61]. Nasal and nasopharyngeal teratomas are developed from the lateral or superior walls and histologically are distinguished based on differentiation of all three germ layers [62]. Nasal obstruction, facial deformity, and feeding difficulties are typical clinical manifestations [63]. Radiological imaging with CT and MRI scans is needed for evaluation of the teratoma’s extension and the sphenoidal bony defect. Intracranial extension is not common; thus, most nasal teratomas can be removed through a transoral approach. However, a craniofacial approach is necessary if an intracranial component exists [64].

Nasal hemangiomas are benign vascular lesions classified into two distinct types: infantile hemangiomas and congenital hemangiomas. The second type is less common, presenting at birth as a fully formed lesion arising from dysregulated angioblast activity during embryogenesis, which leads to localized overgrowth of vascular tissue in the nasal region [65]. Congenital hemangiomas, unlike infantile hemangiomas, do not exhibit rapid growth postnatally [66]. Among congenital hemangiomas, nasal presentations are exceedingly rare, making their true incidence unknown. Congenital nasal hemangiomas are commonly found on the nasal tip or dorsum [7]. They clinically present as soft, compressible, violaceous, or red masses with a lobulated appearance located on or inside the nose. Complications include bleeding if the lesion is traumatized. Less often, nasal obstruction and nasal cartilage aplasia may occur due to the mass effect [67]. Early management of nasal hemangiomas is encouraged, and various modalities have been reported, including observation, systemic and intralesional corticosteroids, propranolol, cryotherapy, surgery, and laser treatment. There is no consensus regarding the optimal treatment algorithm, but a multimodality approach is recommended. Most authors suggest early initiation of medical treatment during the proliferation phase, followed by laser therapy or other more invasive options if necessary [68].

*Nostril polyp* is an exceptionally uncommon congenital nasal anomaly that can manifest as a soft, benign mass arising from the nasal cavity, potentially visible at or near the nostrils. Though polyps are more commonly associated with acquired conditions (like inflammation or chronic sinus disease), congenital nasal polyps have been reported even as a single finding [69], or as part of congenital syndromes, like Pai syndrome [70]. Radiological assessment with a CT scan is an essential part of the diagnostic process. In case of a congenital benign nasal polyp, surgical removal alone may alleviate nasal symptoms, but a high risk of recurrence exists. For that reason, endoscopic sinus surgery is the preferred approach in order to remove the polyp but also open the middle meatus, where the polyp often forms [71].

**Figure 4:**
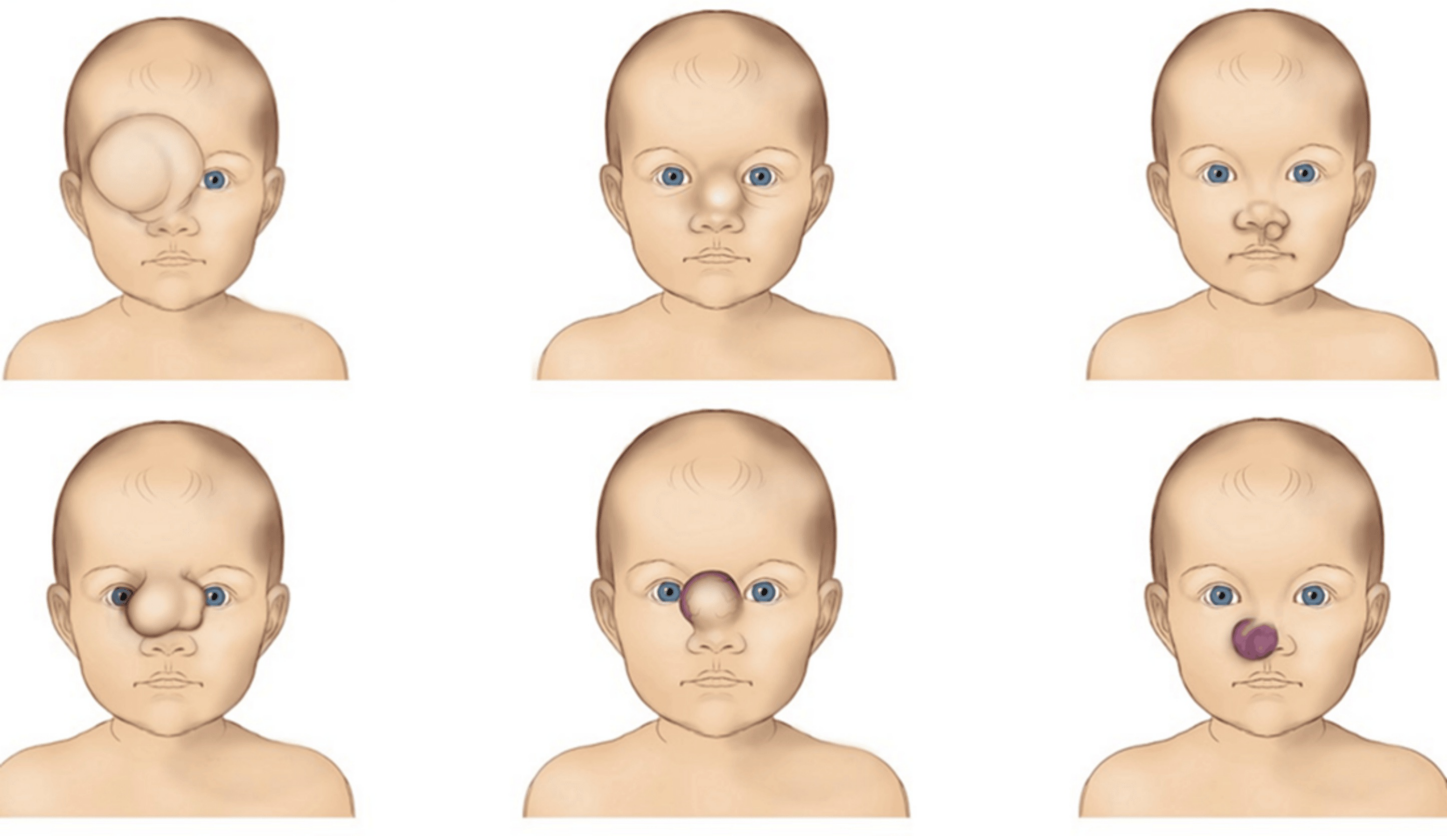
Schematic representation of various Type IV nasal congenital disorders. Image Credit: Vaia Karapepera.

Type IV congenital disorders of the nose are demonstrated in Figure 4.

## Conclusions

Congenital anomalies of the nose, as a complex and rare entity, present diverse challenges. A deep comprehension of nasal embryogenesis is the crucial basis for optimizing patients’ management. Through accurate diagnosis, tailored treatment strategies, and ongoing research efforts, healthcare professionals can ameliorate outcomes and upgrade the quality of life for individuals affected by these anomalies. By addressing both functional and aesthetic concerns, clinicians attempt to restore nasal form and function, improving patients' well-being.

The future of research and clinical management in nasal congenital disorders demands a multidisciplinary perspective in which developmental biology, genetic counseling, and advanced rhino-surgical techniques should be combined. Further investigation of the molecular and genetic mechanisms leading to abnormal embryogenesis of the nose remains critical. On the other hand, the progress in prenatal diagnosis and the potential fetal interventions could be part of a modernized in-utero management.
